# Risk Factors Among Patients with Acute Coronary Syndrome in Rural Kerala

**DOI:** 10.4103/0970-0218.66863

**Published:** 2010-04

**Authors:** Abraham Samuel Babu, Mohammed Haneef, Anupama Naomi Joseph, Manjula Sukumari Noone

**Affiliations:** Department of Rehabilitation, CSI Mission Hospital, Codacal P.O., Tirur – 676 108, Kerala, India; 1 Internal Medicine, CSI Mission Hospital, Codacal P.O., Tirur – 676 108, Kerala, India

Sir,

We found the article by Deb *et al*.(1) both interesting and thought provoking. It was noted that all forms of cardiovascular diseases were included in their study and it was therefore hypothesized that persons with acute coronary syndromes (ACS) may have different risk factors. We report our findings of risk factors among intensive care unit (ICU) patients with ACS from rural Kerala.

A retrospective study of patients admitted to the ICU with ACS during the year 2006-2007 was analyzed. A total of 130 patients were identified from the records. Of these 130 cases, 74.6% (97) were males and 25.4% (33) females with a mean age of 58.4 ± 12.5 years with a range between 24 and 91 years. Of those getting admitted with ACS, 27.9% were above the age of 70 while 66.3% were between the ages of 40 and 60 showing that middle-aged persons were more affected by ACS in this community.

The risk factors [Table 1] for ACS seen in this set-up in rural Kerala were: hypertension (HT), smoking, previous myocardial infarction (MI) and type 2 diabetes mellitus (DM). Alcohol consumption, dyslipidemia and obesity were seen in fewer people. HT was seen to occur in 82.3% of those with ACS. The Seventh Report of the Joint National Committee on Prevention, Detection, Evaluation, and Treatment of High Blood Pressure (JNC 7) classification was used to stratify persons into pre-HT, stage-1 HT and stage-2 HT [Table 1].(2) Most persons in this area were found to have stage-1 HT. Comparison with other studies was not possible due to the variation in classification systems used to grade HT.

**Table 1:** Risk factors for acute coronary syndrome

Risk factors	% (n)
Hypertension	82.3 (107)
Pre-hypertension	19.2 (25)
Stage-1	40 (52)
Stage-2	23.1 (30)
Smoking	50.4 (66)
Previous MI	33.1 (43)
Type 2 DM	33.1 (43)
Controlled	18.5 (24)
Uncontrolled	14.6 (19)
Alcohol	19.2 (25)
Dyslipidemia	18.5 (24)
Obesity	16.2 (21)

MI – myocardial infarction, DM – diabetes mellitus

Smoking was seen to be a major risk factor in 50.4% of the patients. A recent study from West Bengal found that 20.5% of the persons with ischemic heart disease were smokers. (3) The high rate of smokers observed here could be due to lack of awareness on the effects of smoking and its relation to coronary artery disease (CAD) in rural Kerala. (4)

Previous MI and type 2 DM were found to occur among 43/130 persons. Of the 33.1% with type 2 DM, 18.5% had it under control with appropriate measures. The number of persons with type 2 DM and CAD was higher in this area than those reported from other parts of India. (1,5,6)

A recent study by Gupta *et al*.,(7)found a low prevalence of multiple risk factors among young people which increased considerably between the age of 30-39 years. In this sub-group of persons admitted to the ICU, the high rates of risk factors were mainly seen between the ages of 40-49 years. As this age group was not studied by Gupta *et al*.,(7) it can only be assumed that a similar rising trend of risk factors is present in this area as well.

In conclusion, different parts of India seem to demonstrate the same risk factors, but in varied proportions. This sample is not a true representation of the community burden as only cases admitted to the hospital were included. Therefore, large-scale studies covering more geographical areas will be necessary to investigate the risk factors of a particular area and the required preventive measures needed to reduce the prevalence of ACS. Patient education and awareness programs are of great importance in the rural areas to help reduce the burden of ACS.
